# The importance of gut microbiome in the perinatal period

**DOI:** 10.1007/s00431-024-05795-x

**Published:** 2024-10-03

**Authors:** Giulia Catassi, Sandra Garcia Mateo, Annamaria Sara Occhionero, Chiara Esposito, Valentina Giorgio, Marina Aloi, Antonio Gasbarrini, Giovanni Cammarota, Gianluca Ianiro

**Affiliations:** 1 Department of Translational Medicine and Surgery, Università Cattolica del Sacro Cuore, Largo A. Gemelli 8, 00168 Rome, Italy; 2 Pediatric Gastroenterology and Liver Unit, Umberto I Hospital, Sapienza University of Rome, Rome, Italy; 3 Department of Gastroenterology, Lozano Blesa University Hospital, 50009 Zaragossa, Spain; 4 Department of Medical and Surgical Sciences, UOC Gastroenterologia, Fondazione Policlinico Universitario Agostino Gemelli IRCCS, Rome, Italy; 5 Department of Medical and Surgical Sciences, UOC CEMAD Centro Malattie Dell’Apparato DigerenteMedicina Interna E Gastroenterologia, Fondazione Policlinico Universitario A. Gemelli IRCCS, Rome, Italy; 6 Department of Woman and Child Health and Public Health, UOC Pediatria, Fondazione Policlinico Universitario A. Gemelli IRCCS, Rome, Italy

**Keywords:** Neonatal microbiome, Perinatal period, Human health

## Abstract

This narrative review describes the settlement of the neonatal microbiome during the perinatal period and its importance on human health in the long term. Delivery methods, maternal diet, antibiotic exposure, feeding practices, and early infant contact significantly shape microbial colonization, influencing the infant’s immune system, metabolism, and neurodevelopment. By summarizing two decades of research, this review highlights the microbiome’s role in disease predisposition and explores interventions like maternal vaginal seeding and probiotic and prebiotic supplementation that may influence microbiome development.

*Conclusion*: The perinatal period is a pivotal phase for the formation and growth of the neonatal microbiome, profoundly impacting long-term health outcomes.

**Table:** 

**What is Known::** *• The perinatal period is a critical phase for the development of the neonatal microbiome, with factors such as mode of delivery, maternal diet, antibiotic exposure, and feeding practices influencing its composition and diversity, which has significant implications for long-term health.* *• The neonatal microbiome plays a vital role in shaping the immune system, metabolism, and neurodevelopment of infants.*	
**What is New:** *• Recent studies have highlighted the potential of targeted interventions, such as probiotic and prebiotic supplementation, and innovative practices like maternal vaginal seeding, to optimize microbiome development during the perinatal period.* *• Emerging evidence suggests that specific bacterial genera and species within the neonatal microbiome are associated with reduced risks of developing chronic conditions, indicating new avenues for promoting long-term health starting from early life.*

**What is Known::**

*• The perinatal period is a critical phase for the development of the neonatal microbiome, with factors such as mode of delivery, maternal diet, antibiotic exposure, and feeding practices influencing its composition and diversity, which has significant implications for long-term health.*

*• The neonatal microbiome plays a vital role in shaping the immune system, metabolism, and neurodevelopment of infants.*

**What is New:**

*• Recent studies have highlighted the potential of targeted interventions, such as probiotic and prebiotic supplementation, and innovative practices like maternal vaginal seeding, to optimize microbiome development during the perinatal period.*

*• Emerging evidence suggests that specific bacterial genera and species within the neonatal microbiome are associated with reduced risks of developing chronic conditions, indicating new avenues for promoting long-term health starting from early life.*

## Introduction

The perinatal period, spanning from the 22nd week of gestation to the 7th day postpartum, represents a critical window in the development of a neonate [1]. This is a critical phase that lays the groundwork for lifelong health and well-being. The establishment and maturation of the neonatal microbiome—the community of microorganisms that inhabitates our body—during this vital period play a pivotal role in influencing several physiological and immunological pathways [2, 3]. In this period, newborns are highly vulnerable to environmental exposures, making the initial microbial colonization a process of key importance in shaping the neonate’s immune system [4], metabolism, and even neurodevelopment [5, 6]. Mother-related factors, including diet [7], general health condition [8], and use of antibiotics [9], play a significant role in shaping the microbiome of newborns. Also, the mode of delivery—vaginal or cesarean—has been identified as a crucial determinant of microbial composition, with each mode imparting a distinct microbial profile to the neonate [9]. Breastfeeding further enriches the infant’s microbiome, providing a source of beneficial bacteria along with the prebiotic components necessary for their growth [10]. Finally, environmental exposures, including the immediate surroundings and broader ecological context, also contribute to the microbial diversity essential for immune calibration. Early interactions with siblings, pets, and a variety of microorganisms present in the home environment are instrumental in this process [11]. Additionally, prenatal exposures, such as maternal stress and antibiotic usage, have been recently identified as potential influencers of the in utero environment, affecting the microbiome even before birth [12]. Understanding the role of the microbiome in predisposition to diseases and exploring potential interventions aimed at optimizing microbiome development during this crucial period are essential for human health, and intervention strategies aimed at optimizing microbial profiles during these initial stages of life are needed. This review aims to underscore the significance of the microbiome during the perinatal period and elucidate how various factors, including the mode of delivery, maternal diet, antibiotic exposure, gestational age, impact its composition and consequently infant health outcomes.

## Overview of microbiome development during pregnancy and birth

### Microbiome and its relevance in human health 

The gut microbiome represents the complex ecosystem of microorganisms, such as bacteria, viruses, fungi, and protozoa, inhabiting the human gastrointestinal tract. It is incredibly diverse, hosting hundreds to thousands of different microbial species [13]. Every person’s microbiome is distinct, influenced by factors such as genetics, diet, lifestyle, and the surrounding environment [14]. Despite these differences, there are common patterns in the microbial composition among healthy individuals. The gut microbiome is primarily composed of bacteria, with the phyla Firmicutes and Bacteroidetes constituting the bulk of the microbial community. Additional important phyla are Actinobacteria, Proteobacteria, and Verrucomicrobia [11]. The diversity within the microbiome is essential for its function, as each microorganism contributes to health and homeostasis in specific ways. The diversity of the gut microbiome is not static; it evolves throughout an individual’s life, starting from birth [15]. The gut microbiome plays several critical roles in human health: first, it is integral to the development and function of the host’s immune system. It educates the immune system to distinguish between pathogens and non-harmful antigens and can modulate immune responses to reduce the risk of allergic and autoimmune conditions [16]. Microbial components and metabolites interact with immune cells, influencing their function and promoting a balanced immune response [17]. The gut microbiota assists in the digestion of complex carbohydrates, fibers, and proteins that the host cannot digest. It also produces essential vitamins and nutrients, including vitamin K, B vitamins, and short-chain fatty acids (SCFAs), which are critical for gut health, energy production, and regulation/downgrading of intestinal inflammation [18]. Eventually, the gut microbiome competes with pathogenic bacteria for nutrients and attachment sites on the gut wall, a phenomenon known as colonization resistance. It also produces antimicrobial substances and modulates the gut environment to prevent pathogen colonization and infection [19].

### The importance of the gut microbiome in the perinatal period: the native core microbiome

A foundational set of microbial species, known as the native core microbiota, resides within the vast and complex ecosystem of the human gut [20]. This essential group of microorganisms begins to colonize the gut in early life, setting the stage for an individual’s microbiome composition [20]. The native core microbiota plays a critical role in establishing the microbiome’s stability and resilience [21]. Although the microbiome differs among individuals, the existence of a core composition common to all underscores its fundamental role in performing essential functions necessary for human health [22]. The perinatal period serves as a critical window for both immune and metabolic programming, and the native core microbiota plays a central role in this developmental phase. The establishment of the core microbiota in newborns is influenced by a multitude of factors, including but not limited to maternal microbiota, mode of delivery, and diet [23]. Fouhy et al. underscore the significance of these perinatal factors, revealing that these can shape the gut microbiota up to 4 years post-birth, emphasizing the lasting impact of early microbial colonization. Children born full-term exhibited higher microbiota diversity compared to preterm infants, indicating the profound influence of birth circumstances [24]. Fascinatingly, this research suggests that, although breastfeeding and exposure to antibiotics in the first year have initial impacts, these effects might be eclipsed by dietary and environmental factors encountered later on. The significance of nurturing a healthy core microbiota during this period cannot be overstated, as it influences the development of the infant’s immune system, aiding in the differentiation between harmful and benign substances and thus reducing future risks of allergies and autoimmune diseases [25]. Additionally, the core microbiota is involved in metabolic programming, influencing energy balance and metabolic functions that can affect the individual’s susceptibility to metabolic disorders like obesity and diabetes later in life [26]. Moreover, a robust core microbiota is crucial for maintaining the integrity of the gut barrier, which prevents harmful pathogens and substances from entering the bloodstream, thereby safeguarding against infections and systemic inflammation [27]. The perinatal period is marked by a heightened vulnerability in the establishment of the native core microbiota, with potential disruptions posing long-term health implications [28].

### Changes in microbiota during the perinatal period

The development and transformation of the microbiome during the perinatal period are complex processes that are vital for both maternal and neonatal health. This period encompasses microbiological shifts starting from pregnancy through to the postnatal phase, each carrying significant implications for the physiological and immunological outcomes of both the mother and the offspring [29].

#### Microbiome changes during pregnancy

During pregnancy, the maternal microbiome undergoes specific alterations to accommodate and support the developing fetus and to prepare the body for childbirth. Early research indicates a gestation-related microbiome adaptation, initially characterized by an increase in microbial diversity [30]. This diversity is thought to enhance the maternal immune system’s capacity to protect both mother and fetus [31]. As pregnancy progresses, a notable shift towards a microbiota with reduced diversity and increased representation of pro-inflammatory taxa occurs [32]. This adaptation appears to facilitate efficient energy harvest from the diet, an essential factor for fetal growth and development [31]. In exploring the complexities of the microbiome during pregnancy, several studies illuminate its profound alterations and implications for maternal and fetal health. Mallott et al. investigated the role of hormonal mediation in microbiome changes, particularly the significant impact of progestagens on microbial diversity during pregnancy and lactation. Their findings in Phayre’s leaf monkeys highlight a species-specific effect, suggesting that these hormonal influences might be critical for microbiome adaptations in various species [33]. Complementing this species-specific view, Gorczyca et al. provide a broader overview of the human maternal gut microbiome’s evolution during pregnancy [34]; they observe an increase in protective bacteria like *Akkermansia* and *Bifidobacterium*, which contribute to immunological and metabolic health, illustrating the beneficial shifts that occur within the human maternal microbiome [34]. Further extending the discussion, Zakaria et al. highlight the microbiome’s integral role in maintaining pregnancy health through its influence on immunological, endocrinological, and metabolic pathways [35]: the microbiome helps modulate the mother’s immune system to prevent excessive inflammation and protect against infections. This is crucial for avoiding complications such as preterm birth and preeclampsia. The microbiome supports the hormonal environment necessary for placental function, which is vital for the fetus’s nutrient supply; certain bacteria like *Bifidobacteria* grow in response to the higher progesterone levels, supporting overall health. Metabolically, the microbiome aids in the digestion and effective use of nutrients, crucial for fetal development. It also helps regulate insulin sensitivity, which can prevent gestational diabetes [35]. In line with this, Giannella et al. explicitly linked microbiota alterations with pregnancy complications such as hypertensive disorders and gestational diabetes [36]. This suggests that shifts in the microbiome could serve as indicators or contributors to these conditions, underlining the potential for microbiome analysis to inform about pregnancy health risks. Simultaneously, the fetal environment, once thought to be sterile, is now understood to potentially harbor its own unique microbiota, with implications for the immune and metabolic programming of the fetus [37]. Studies have proposed that the placenta, amniotic fluid, and umbilical cord blood might contain microbial communities that could influence the developing fetal immune system, setting the stage for postnatal microbial colonization[37–39].

#### The transition at birth

The delivery is a critical moment for microbial transfer and colonization in the newborn. Birth through vaginal delivery allows the infant to come into contact with the mother’s vaginal and fecal microbiota, promoting the transfer of beneficial microorganisms like *Lactobacillus* and *Bifidobacterium* species to the newborn’s gut [40]. This early microbial seeding plays a pivotal role in shaping the infant’s microbiome and immune system development. Conversely, babies delivered by cesarean section initially acquire microbes from the skin and hospital surroundings, potentially resulting in a different initial composition of the microbiome, with higher abundance of Enterobacteriaceae and Enterococcaceae families, along with depletion of *Bacteroides* genus and a delay in the maturation of gut microbiota in early life [41]. This difference in early microbiome establishment is associated with varied health outcomes, including increased risks for certain immune and metabolic disorders in cesarean-delivered infants.

#### Postnatal microbiome development

After birth, the infant’s microbiome undergoes rapid diversification. The TEDDY study [42] has identified three distinct phases of the gut microbiome’s evolution: The development phase (3–14 months) is characterized by rapid changes in the microbiome composition, heavily influenced by factors such as mode of birth and breastfeeding. In the transitional phase (15–20 months), during this period, the microbiome begins to show signs of settling but is still undergoing significant shifts in its composition. Dietary changes, such as the introduction of solid foods, play a crucial role in this phase. Finally, in the stable phase (31–46 months), the microbiome reaches a more stable and mature state, resembling that of an adult’s microbiome. However, it can still be influenced by the child’s diet and environment. The development of the infant’s microbiome in the first years of life is critical for immune system maturation, with evidence suggesting that early microbial exposures are key to training immune cells to distinguish between harmful and non-harmful antigens [43].

#### Preterm infants

Preterm infants are at heightened risk for complications such as necrotizing enterocolitis (NEC), sepsis, and mortality due to their immature gut microbiota and underdeveloped immune systems [44]. Unlike term infants, preterm babies often experience delayed microbial colonization, lower bacterial diversity, and a higher presence of pathogenic bacteria, which leads to a greater susceptibility to these life-threatening conditions. Probiotics, particularly strains from *Bifidobacterium* and *Lactobacillus*, have shown promising results in helping preterm infants establish a healthier gut microbiome, enhancing gut barrier function and modulating immune responses [45]. Several meta-analyses have demonstrated the efficacy of probiotics in reducing NEC, sepsis, and mortality in preterm infants. A network meta-analysis identified the combination of *Bifidobacterium longum*, *B. bifidum*, *B. infantis*, and *Lactobacillus acidophilus* as significantly reducing NEC (RR 0.31), sepsis (RR 0.47), and mortality (RR 0.26) [46]. Another study emphasized that *Bifidobacterium lactis* was particularly effective in reducing NEC, especially in infants exclusively fed with human milk, as compared to formula-fed infants. The human milk provides oligosaccharides that function as prebiotics, likely enhancing the effects of probiotics in promoting a healthy gut environment and preventing NEC more effectively than formula feeding [47]. The Connection trial represents a significant advancement in this field, as it is the first large-scale, multicenter study to evaluate the use of pharmaceutical-grade probiotics for NEC prevention in preterm infants. This Phase 3 randomized, double-blind, placebo-controlled trial is investigating the efficacy and safety of the probiotic strain IBP-9414 in preterm infants weighing 500 to 1500 g. The trial, involving over 2000 infants, is a milestone in probiotic research because it uses probiotics that are industrially produced with pharmaceutical-level quality standards, ensuring consistent dosing and safety. While the full results are yet to be published, the trial’s completion will provide valuable insights into the role of probiotics, specifically IBP-9414, in preventing NEC and other negative outcomes related to prematurity, such as sepsis and mortality. This represents a crucial step forward in translating probiotic research into clinical practice with the potential to improve outcomes for preterm infants worldwide [48].

Moreover, recent evidence from a Bayesian network meta-analysis further supports the beneficial effects of probiotic combinations, such as *B. lactis*, *B. longum*, and *L. acidophilus*, in reducing NEC and mortality. These findings highlight the critical importance of early probiotic administration to promote healthy microbiome development, particularly in very low birth weight (VLBW) infants in low- and middle-income countries [49].

## Factors influencing early microbiome colonization

**Fig. 1:**
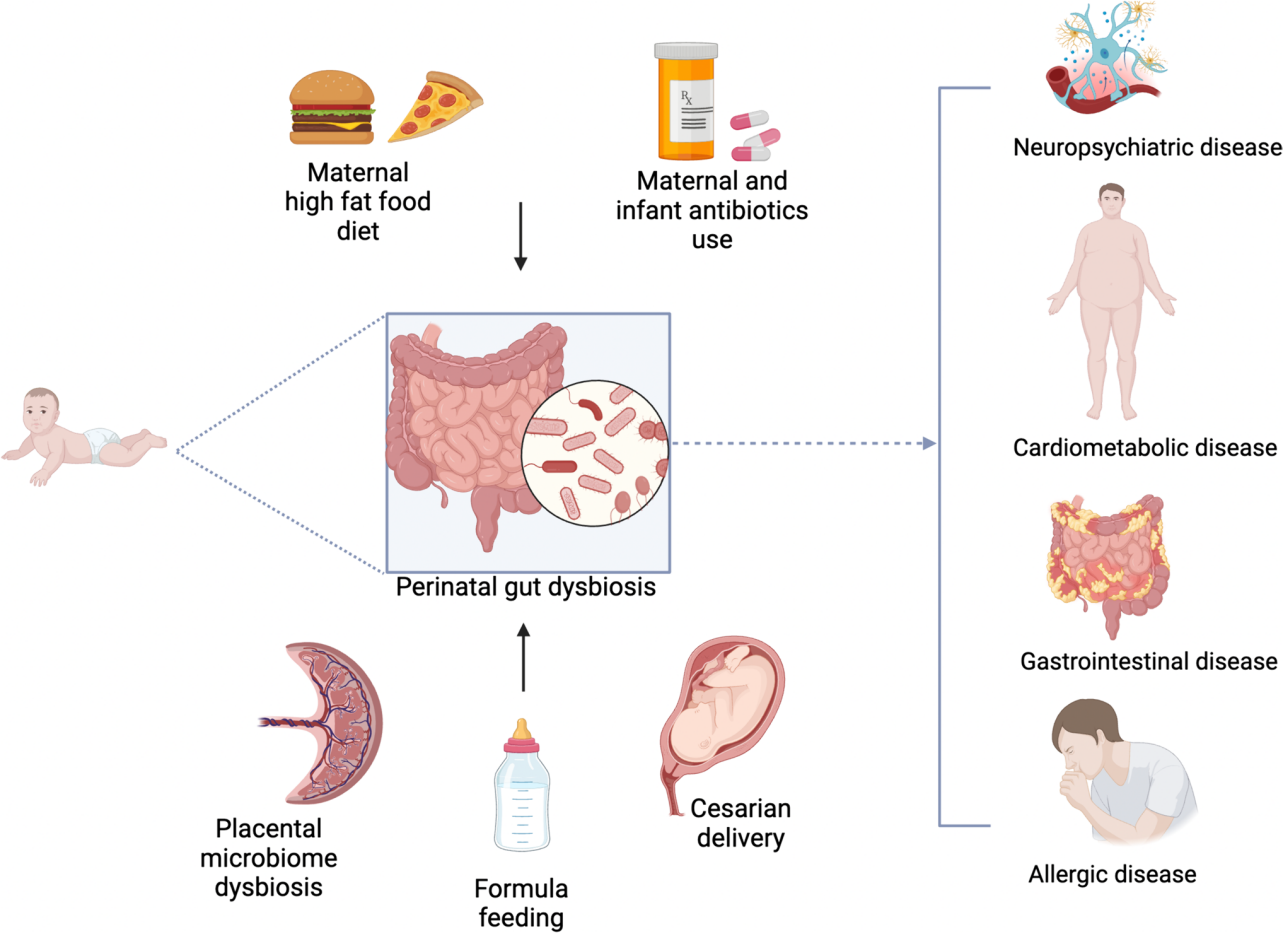
Factors influencing early microbiome colonization

The early colonization and development of the human microbiome are influenced by a complex interplay of factors that begin at birth and continue through infancy [50]. Additionally, the environment, including the use of antibiotics in infancy and exposure to siblings or pets, plays a significant role in diversifying and shaping the microbial community [6]. Figure 1 offers an in-depth visual summary of these diverse factors, demonstrating how they intricately interact and impact the foundational development of microbial communities.

### Maternal diet

The intricate relationship between maternal diet and the development of the infant gut microbiota underscores the profound impact maternal nutrition has on infant health during pregnancy and lactation. Higher maternal consumption of fruits and vegetables is associated with a more diverse and beneficial microbial composition in the infant gut, especially in those who are delivered vaginally and exclusively breastfed [51, 52]. This dietary pattern is linked to increased counts of beneficial bacteria such as *Cutibacterium*, *Parabacteroides*, and *Lactococcus*, along with reduced counts of potentially pro-inflammatory bacteria [53].

Mothers with a high intake of fibers and plant proteins tend to have breast milk enriched with beneficial bacteria like *Bifidobacterium* [54].

The broader relationship between diet quality during pregnancy—including the intake of fiber, fat, and vitamins—and the shaping of both maternal and infant gut microbiomes is further corroborated by various studies [51, 55]. Although specific dietary components like salmon may not directly impact the gut microbiota of mothers and infants, factors such as maternal weight gain during pregnancy and the mode of infant feeding are significant [56]. Obesity in mothers, often associated with poor dietary patterns, correlates with shorter breastfeeding durations and potentially impacts the infant’s microbiome development and health [57].

The systematic review by Maher et al. [55] consolidates these observations, highlighting the association between maternal diet—particularly the consumption of high-fat diets and fiber—and the gut microbiota composition in both mothers and infants.

### Placental microbiota

Studies utilizing advanced molecular techniques have identified the presence of a low-biomass but diverse microbial community in the placenta [39]. This microbiota resembles the oral microbiome more closely than those of the gut or vagina, suggesting hematogenous spread from the oral cavity to the placenta [38]. Microorganisms detected in the placenta include a variety of bacteria, such as Firmicutes, Tenericutes, Proteobacteria, Bacteroidetes, and Fusobacteria [58]. The presence of these microbes in the placenta challenges the sterile womb paradigm and suggests a mechanism for early microbial colonization of the fetus. The discovery of the placental microbiota has led to speculation about its role in shaping the fetal immune system and microbiome [59]. It has been hypothesized that the placental microbes could prime the fetal immune system, introducing the fetus to non-pathogenic antigens and helping to develop tolerance. This early microbial exposure is crucial for the proper maturation of the fetal immune system, potentially influencing the child’s susceptibility to allergies, autoimmune diseases, and other immune-related conditions later in life [60].

Furthermore, the placental microbiota might contribute to the initial seeding of the fetal gut microbiome [39]. Microbes from the placenta could be swallowed by the fetus, colonizing the gut and setting the stage for further microbial colonization after birth. This early colonization is essential for the development of a healthy gut microbiome, which plays a critical role in nutrient absorption, metabolism, and protection against pathogens [38]. The existence and role of the placental microbiota have significant implications for fetal microbiome development. It suggests that the foundations for the gut microbiome, and by extension the immune system, are laid before birth. This understanding underscores the importance of maternal health and diet, as these factors could influence the composition of the placental microbiota [39].

### Antibiotic exposure

The administration of antibiotics to both mothers during pregnancy or labor and to infants shortly after birth has been identified as a significant factor that can alter the neonatal gut microbiota [61].

#### Maternal antibiotic exposure

When antibiotics are administered during pregnancy, they can cross the placental barrier and reach the fetal environment [62]. Antibiotics are designed to kill or inhibit the growth of bacteria, and they do not discriminate between harmful pathogens and beneficial microbial communities. Thus, maternal antibiotic use can disrupt the maternal microbiota, which in turn can affect the microbial populations available for vertical transmission to the fetus [62]. This disruption can lead to a reduction in microbial diversity and a delay in the colonization of beneficial microbes in the neonate [61].

The alteration of the maternal microbiota and its effects on the fetal microbiome may have implications for the development of the fetal immune system [63]. A diverse microbiome is essential for the proper training of the immune system, teaching it to differentiate between harmful pathogens and benign antigens. Disruptions in early microbial exposure due to antibiotic use can increase the risk of developing conditions such as allergies, asthma, and autoimmune diseases in later life [64].

#### Neonatal antibiotic exposure

Antibiotics administered directly to infants can have a profound impact on their nascent microbiota [65]. The early postnatal period is a critical time for microbial colonization and the establishment of a stable gut microbiome. Antibiotics during this period can cause significant disruptions, including decreased microbial diversity, delayed colonization by beneficial microbes, and an increased abundance of antibiotic-resistant bacteria [66]. These early disruptions in the gut microbiota have been associated with an increased risk of diseases in both infancy and later life [67]. Conditions such as necrotizing enterocolitis (NEC) in preterm infants, obesity, type 1 diabetes, and inflammatory bowel disease (IBD) have been linked to alterations in the early-life microbiome [68]. The use of antibiotics can also impact the development of the gut-brain axis, potentially influencing neurodevelopment and behavior[69].

### Mode of delivery

The delivery method significantly influences the early colonization of the neonatal microbiome, displaying notable disparities between infants delivered through cesarean section (C-section) and those born vaginally [70]. This initial establishment of the microbiome is vital in determining the infant’s health and development in the future [71].

#### Microbiome colonization in vaginal delivery

Infants born through vaginal delivery are exposed to their mother’s vaginal and fecal microbiota. This exposure is a critical process that initiates the colonization of the infant’s gut with microbes, including *Lactobacillus*, *Prevotella*, and *Bacteroides* [72]. These bacteria are essential for the development of the infant’s immune system and metabolic processes. The early colonization by these microbes helps in the maturation of the immune system, training it to distinguish between harmful and benign antigens and reducing the risk of allergies, autoimmune diseases, and metabolic disorders [73].

#### Microbiome colonization in cesarean section

In contrast, infants born via C-section are exposed to a different microbial environment, primarily consisting of skin-associated bacteria such as *Staphylococcus*, *Corynebacterium*, and *Propionibacterium* [74]. This difference in early microbial exposure can lead to a delay in the colonization of the gut by the beneficial microbes typically acquired through vaginal delivery [41]. The altered microbiome composition in C-section-delivered infants is associated with a higher risk of developing a range of health issues, including allergies, obesity, asthma, celiac disease, and type 1 diabetes [75–78]. The lack of exposure to maternal vaginal and fecal microbes is thought to impact the development of the infant’s immune system, potentially leading to an increased susceptibility to these conditions [3]. A systematic review by Princisval et al. indicated that CD-born infants exhibit lower abundance of *Bifidobacterium* and *Bacteroides* species up to 18 months of age, regardless of whether the CD was performed before or after labor onset, challenging the notion that the absence of *Bacteroides* is solely due to lack of exposure to the vaginal canal [79, 80]. Studies also suggest that breastfeeding can mitigate these effects, contributing to the construction and stabilization of gut microbiota in CD-delivered infants [81, 82]. Liu et al. [83] further demonstrated that exclusive breastfeeding partially restores the perturbed gut microbiota in C-section infants, underscoring the importance of feeding patterns in these cases.

### Feeding practices

Feeding practices play a critical role in the colonization and development of the infant’s microbiome, with breastfeeding and formula feeding having distinct impacts [84]. These differences in early nutrition can influence the infant’s health both in the short term and throughout their life.

#### Breastfeeding and the infant microbiome

Breastfeeding is recognized for its unparalleled benefits in supporting the development of a healthy infant microbiome [85]. Human breast milk is not only a source of essential nutrients but also contains a variety of bioactive components, including antibodies, oligosaccharides, and a complex ecosystem known as the human milk microbiome (HMM), which contains beneficial bacteria, viruses, fungi, and other microorganisms [86, 87]. These components play a crucial role in shaping the infant’s gut microbiome. Breastfed infants typically have a gut microbiome dominated by beneficial bacteria such as *Bifidobacterium* and *Lactobacillus* [88, 89]. These bacteria are adept at fermenting the human milk oligosaccharides (HMOs) found in breast milk, producing SCFAs that have a critical role in maintaining gut health, modulating the immune system, and protecting against pathogen colonization [43]. The dynamic nature of breast milk also allows for the vertical transmission of microbiota from mother to infant through breastfeeding, seeding the infant’s gut and oral microbiota with pioneer bacteria that aid in immune system maturation [87]. Moreover, this microbial diversity is influenced by maternal factors such as diet, mode of delivery, and geographic location, which may affect the composition and richness of the milk’s microbiome. The interaction between the beneficial microbes and the immunological components in breast milk is critical in training the infant’s immune system to recognize harmful versus benign antigens, potentially reducing the risk of allergies and autoimmune diseases later in life [90].

#### Formula feeding and the infant microbiome

Formula feeding, while providing a vital alternative when breastfeeding is not possible, results in a different microbiome composition compared to breastfed infants. Formula-fed infants tend to have a more diverse microbiome earlier on, with higher proportions of bacteria such as *Clostridium* and *Bacteroides* [91]. The diversity and composition of the microbiome in formula-fed infants can resemble a more mature microbiome but may lack the specific beneficial bacteria that are fostered by the unique components of breast milk [92]. The absence of HMOs and other bioactive molecules in formula can lead to a different pattern of microbial colonization and metabolic activity in the gut [93]. The differences in microbiome development between breastfed and formula-fed infants have been linked to variations in health outcomes. Formula-fed infants may have an increased risk of developing conditions such as obesity, type 2 diabetes, and gastrointestinal infections [94–96]. The altered microbial exposure and the absence of breast milk’s immunological protection can influence the infant’s immune system development and metabolic programming. Efforts to bridge the gap between breast milk and formula have led to the development of enriched formulas containing HMOs, probiotics, prebiotics, and postbiotics, aiming to emulate the gut microbiome of breastfed infants and support healthy immune development [95, 97]. Several studies have demonstrated that the gut microbiota composition of infants consuming modern formulas enriched with prebiotics resembled more closely that of breastfed infants [98, 99], with increased relative abundances of *Bifidobacterium* and decreased relative abundances of Enterobacteriaceae and Peptostreptococcaceae [100]. Despite these advancements, breastfeeding remains unparalleled in its role in early microbial acquisition and community succession in infants.

## Established links between perinatal microbiome and health

**Fig. 2:**
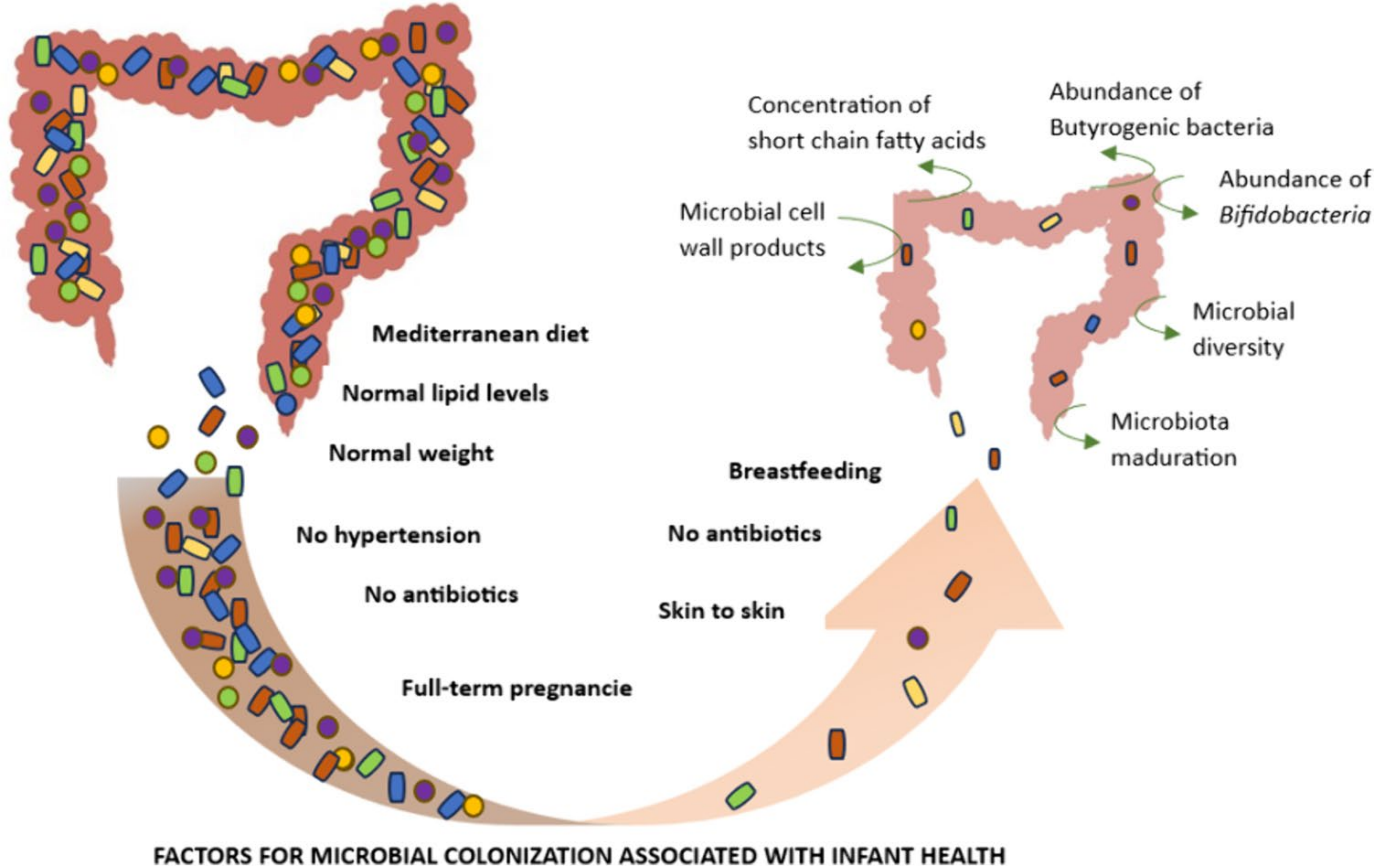
Established links between perinatal microbiome and health

An increasing body of evidence underscores the pivotal role of early microbiome composition in the development of some diseases, highlighting a complex interplay between genetic predisposition, environmental factors, and microbial ecology. Figure 2 provides a detailed visual representation of the diseases associated with altered microbiota, as well as the factors that influence these alterations.

### Cardiometabolic disorders

Recent research suggests a connection between the perinatal microbiome and the risk of developing cardiometabolic disorders, including obesity, diabetes, and other metabolic conditions [101]. This body of evidence highlights the profound impact of early microbial exposures on the metabolic programming of the infant, with long-term implications for health and disease [102, 103].

#### Obesity

The relationship between the early-life microbiome and obesity is one of the most compelling areas of study within the realm of cardiometabolic health. Infants with a reduced diversity of gut microbiota or specific imbalances in their microbial composition are at an increased risk of becoming overweight or obese later in life [104, 105]. Specific maternal dietary patterns have been associated with shaping the neonatal gut microbiota, predisposing infants to overweight during the first 18 months of life. For instance, early dominance of certain bacterial species, such as *Eubacterium rectale* from the Firmicutes phylum, over others like *Bacteroides thetaiotaomicron*, belonging to the phylum of Bacteroidetes, has been linked to increased energy harvest from the diet, contributing to weight gain and adiposity [106, 107]. Factors like cesarean section [108], formula feeding [108, 109], early-life antibiotics [108, 110, 111], and maternal overweight/obesity [112–115] influence the development of an “obese-type” microbiota [116], characterized by an abundance of pro-inflammatory Lachnospiraceae, including *Coprococcus*, *Ruminococcus*, and *Blautia* [117] and a decrease in protective *Bifidobacteria* and *Bacteroidetes*, with *Bacteroides fragilis* being a prime example [108]. Breastfeeding has been associated with protective effects against childhood overweight and obesity, as well as type 2 diabetes, via the gut microbiota. A meta-analysis also revealed a dose–response relationship, indicating that an increased duration of breastfeeding is associated with a lower risk of becoming overweight [105].

#### Other metabolic conditions

The early-life microbiome also appears to play a role in other metabolic conditions, such as metabolic syndrome and insulin resistance, which are precursors to more severe diseases like type 2 diabetes and cardiovascular disease [118]. Maternal obesity may introduce microbiota aberrations relevant for metabolic health through direct vertical microbiota transfer and priority effects, leading to intergenerational effects and the development of metabolic disorders [119]. The mechanisms underlying these associations involve the microbiome’s influence on inflammation, energy metabolism, and insulin sensitivity [120]. For example, specific microbial metabolites, such as SCFAs, have been shown to exert beneficial effects on host metabolism, including enhancing insulin sensitivity and regulating appetite and energy expenditure [121]. Disruptions in the production of these metabolites due to an imbalanced microbiome can contribute to the pathophysiology of metabolic disorders [122].

### Allergies

The intricate relationship between the early microbiome composition and the susceptibility to allergic conditions has garnered substantial attention in recent research, underscoring the pivotal role of microbial exposures in infancy in shaping immune responses to allergens. This body of research elucidates how variations in the diversity and specific compositions of the microbiome during the perinatal period significantly influence the risk of developing allergies, such as eczema, asthma, and food allergies [123]. Central to this discussion is the concept that the early microbial environment is fundamental in guiding the immune system towards a path of immune tolerance, essentially teaching it to differentiate between benign substances and genuine pathogens, thereby reducing inappropriate immune reactions that manifest as allergies [124].

Studies consistently demonstrate a correlation between reduced microbial diversity and decreased microbiota maturation in the gut microbiome of infants within their first months of life and an elevated risk of allergy development [125, 126]. This appears to limit the immune system’s capacity to establish tolerance. Furthermore, research has pinpointed specific bacterial genera, including *Faecalibacterium*, *Akkermansia*, and *Bifidobacterium*, whose presence or abundance is inversely related to allergy risk [127, 128]. In contrast, a heightened presence of bacteria such as *Clostridium* or *Staphylococcus* is associated with an increased allergy risk, suggesting that the composition of the early-life microbiome is a critical determinant of allergic disease susceptibility [127].

The intricate relationship between the early microbiome composition and the susceptibility to allergic conditions has garnered substantial attention in recent research, underscoring the pivotal role of microbial exposures in infancy in shaping immune responses to allergens. This body of research elucidates how variations in the diversity and specific compositions of the microbiome during the perinatal period significantly influence the risk of developing allergies, such as eczema, asthma, and food allergies [117]. The early microbial environment is fundamental in guiding the immune system towards a path of immune tolerance, essentially teaching it to differentiate between benign substances and genuine pathogens, thereby reducing inappropriate immune reactions that present as allergies [118].

Studies consistently demonstrate a correlation between reduced microbial diversity and decreased microbiota maturation in the gut microbiome of infants within their first months of life and an elevated risk of allergy development, limiting the immune system’s capacity to establish tolerance [119, 120]. Furthermore, research has pinpointed specific bacterial genera, including *Akkermansia*, and *Bifidobacterium*, whose presence or abundance is inversely related to allergy risk [121, 122]. In contrast, a heightened presence of bacteria such as *Clostridium* or *Staphylococcus* is associated with an increased allergy risk, suggesting that the composition of the early-life microbiome is a critical determinant of allergic disease susceptibility [121].

Recent studies, such as those by De Filippis et al. [129], further expand our understanding by identifying specific microbiome features associated with pediatric allergies and their potential impact on immune tolerance mechanisms. Allergic children exhibit a distinct gut microbiome marked by an increased abundance of *Ruminococcus gnavus* and *Faecalibacterium prausnitzii* and a decreased presence of *Bifidobacterium longum* and fiber-degrading taxa like *Bacteroides dorei* and *Bacteroides vulgatus*. These microbial signatures not only correlate with higher pro-inflammatory potential—evidenced by enriched genes related to bacterial lipopolysaccharides and urease—but also predict the acquisition of immune tolerance, underscoring their role in allergic disease progression and resolution.

Vaginally delivered and breastfed infants typically exhibit a microbiome composition that is associated with a lower risk of allergies, highlighting the critical impact of these early-life factors on the maturation of the immune system [130, 131]. Additionally, the use of antibiotics during infancy has been shown to disrupt the gut microbiome, reducing microbial diversity and altering the balance of microbial communities [132]. This disruption is linked to an increased incidence of allergic diseases, further emphasizing the importance of preserving microbial diversity for immune system development. The mechanisms by which the early-life microbiome influences allergy development are multifaceted, involving the regulation of immune function through microbial metabolites like SCFAs [133]. These metabolites are instrumental in promoting the development of regulatory T cells, crucial for maintaining immune tolerance. Moreover, the early microbiome plays a significant role in reinforcing the gut barrier, preventing the translocation of allergens, and fostering a healthy immune response to dietary antigens [16].

### Gastrointestinal disorders

The early microbiome plays a pivotal role in the development and maturation of the gastrointestinal (GI) tract, influencing immune responses and the integrity of the gut barrier [134]. Disturbances in the early microbiome are increasingly recognized for their potential to predispose individuals to a range of gastrointestinal disorders, including infant colic, celiac disease, and inflammatory bowel disease (IBD) [135]. These conditions highlight the critical importance of microbial exposures in the perinatal period for long-term gastrointestinal health.

#### Infant colic

Infant colic, characterized by excessive crying and discomfort in otherwise healthy infants, has been linked to alterations in the gut microbiota [136]. Studies have shown differences in the microbiome composition of colicky infants compared to their non-colicky peers, including lower diversity and variations in the abundance of specific bacterial groups [137]. For instance, colicky infants often have fewer *Lactobacilli* and an increased presence of gas-producing bacteria, which might contribute to gastrointestinal discomfort and the symptoms of colic [138]. These findings suggest that interventions aimed at modulating the gut microbiota, such as the use of probiotics, could offer potential benefits in managing colic symptoms [139].

#### Celiac disease

Celiac disease is an autoimmune disorder triggered by the ingestion of gluten in genetically susceptible individuals [140]. Emerging evidence suggests that early-life microbial exposures can influence the risk of developing celiac disease [141]. Factors such as mode of delivery and antibiotic use have been associated with alterations in the gut microbiome that may affect immune tolerance to dietary gluten [142]. This does not hold true for breastfeeding for which a prospective study indicated that breastfeeding does not serve as a protective factor against the development of celiac disease [143]. Additionally, the timing of gluten introduction and the composition of the infant’s microbiome at this critical period might interact to influence the development of celiac disease [144]. Research indicates that a balanced and diverse microbiome may play a protective role, potentially reducing the risk or delaying the onset of the disease.

#### Inflammatory bowel disease (IBD)

IBD, that include Crohn’s disease and ulcerative colitis, are characterized by chronic inflammation of the gastrointestinal tract [145]. The pathogenesis of IBD is multifactorial, involving genetic susceptibility, environmental factors, and dysregulation of the immune system, with the gut microbiome playing a central role [145]. Early microbiome disturbances, such as reduced microbial diversity or imbalances in specific microbial communities, have been implicated in the development of IBD [146]. These disturbances can impact immune system development, gut barrier function, and the balance of pro-inflammatory and anti-inflammatory signals in the gut [147]. The result is an increased susceptibility to inappropriate immune responses against the gut microbiota, contributing to the chronic inflammation observed in IBD [148].

### Neuropsychiatric diseases

The emerging research on the gut-brain axis during the perinatal period is shedding light on potential of early microbiome composition to influence neuropsychiatric outcomes from infancy into later life [149]. The concept of the gut-brain axis encompasses the intricate communication network that links the gastrointestinal tract and the brain [150]. During the perinatal period, this axis is particularly influential, as it is a time when both the gut microbiota and the central nervous system are undergoing rapid development and maturation. The initial colonization of the gut microbiome, influenced by factors such as mode of delivery, maternal diet, and early feeding practices, plays a crucial role in this process [151]. These early microbial communities can produce various compounds, including neurotransmitters and inflammatory mediators, that can cross the placental barrier and potentially influence fetal brain development [152].

#### Early microbiome composition and neuropsychiatric disorders

Research has begun to explore the association between disruptions in the early microbiome and the risk of neuropsychiatric disorders such as autism spectrum disorder (ASD). Imbalances in the perinatal microbiome could impact neurodevelopmental processes, affecting neural circuitry and leading to long-term changes in behavior and cognitive function [153]. Alterations in both the salivary and gut microbiomes in children with ASD suggest a unique microbial composition, which could be implicated in the disorder’s pathogenesis through immune and inflammatory responses [154, 155]. They are characterized by an increased abundance of genera such as *Faecalibacterium*, *Rothia*, *Bacteroides*, *Oribacterium*, *Lachnoanaerobaculum*, and *Megasphaera*, as well as families including Micrococcaceae, Ruminococcaceae, Bacteroidaceae, and Lachnospiraceae. Conversely, there is a decreased abundance of genera like *Pseudomonas* and *Abiotrophia*, families such as Pseudomonadaceae and Aerococcaceae, the order Pseudomonadales, and the species *Pseudomonas graminis* in children with ASD [154, 155]. This microbial profile is correlated with neurodevelopmental outcomes, indicating a direct link between specific microbial profiles and the manifestation of ASD. Later, other early-life shifts in gut microbiota composition in infants at elevated risk of ASD have been identified [156]. These infants exhibited a lower abundance of *Bifidobacterium* and a higher presence of *Clostridium*-related species, along with a decrease in gamma-aminobutyric acid (GABA) in fecal samples [157]. Such microbial differences were associated with developmental changes in language skills, underscoring the significant role of gut microbiota in neuro-immune modulation from an early age [158]. Another study indicates that maternal diet high in inflammatory components can prime the neurodevelopmental trajectory of the offspring towards disorders like ASD [159], highlighting the role of maternal immune activation and neuroinflammation, which are significantly influenced by maternal dietary patterns [160]. These inflammatory responses may lead to altered neurodevelopmental outcomes in the offspring, echoing the patterns observed in the microbial alterations associated with ASD and language development [159]

## Potential for therapy

### Vaginal seeding

As already discussed, the infant gut microbiome is significantly influenced by the mode of delivery [70]. To mitigate this influence, strategies such as maternal vaginal seeding have been recently explored. Maternal vaginal seeding involves swabbing cesarean-born infants with maternal vaginal fluids to potentially lessen the differences in microbiota development between vaginally born infants and those born by cesarean section [161]. In a recent randomized controlled trial, vaginally seeding neonates delivered by cesarean section resulted in a partial restoration of their microbiota, similar to that of their vaginally delivered counterparts, with increased transmission of microbiota from mother to child and compositional changes in the microbiota of their skin and stool [162]. However, a different study cast doubt on the contribution of the mother’s vaginal microbiota to the microbiota that is seeded in the baby’s mouth and nose and proposed the possibility that other sources, such breast milk, may play a role in this process [163]. Despite the perceived benefits, a recent study raised concerns about the unproven benefits and potential risks to neonatal health associated with vaginal seeding, advocating for the confinement of the procedure to carefully monitored clinical trials [164].

In conclusion, while the concept of vaginal seeding is intriguing, current evidence remains inconclusive, and it cannot be recommended as a standard practice until more conclusive research is conducted.

### Prebiotics and probiotics

Probiotic supplementation during pregnancy, particularly with *Lactobacillus rhamnosus GG*, has been shown to positively impact the maternal vaginal microbiota [165]. This intervention significantly reduced the presence of pathogenic microorganisms, thereby laying the groundwork for a healthier microbial environment for the infant. However, there is limited evidence that this intervention influences the overall diversity of the infant microbiome or determines sustained colonization [166, 167]. Initial neonatal supplementation with *Lactobacillus rhamnosus GG* can increase the abundance of beneficial *Bifidobacteria* in the infant’s gut, a hallmark of a healthy gut microbiome [168], while further research has not shown a clear link between probiotic administration and higher concentrations of *Bifidobacterium* in the feces [169, 170].

Among prebiotics, human milk oligosaccharides (HMOs) are a cornerstone in nurturing a healthy infant gut microbiome. The advantages of breast milk have been mimicked in baby formulas by adding these oligosaccharides such as lacto-N-neotetraose and 2′-fucosyllactose [171]. HMOs are instrumental in promoting the growth of beneficial bifidobacteria, such as *Bifidobacterium longum*, *B. bifidum*, and *B. longum subspecies infantis*. These specific strains are known for their immunogenic effects that are crucial in the early stages of life [172]. These formula adaptations have shown promising results, enhancing the production of lactate and SCFAs.

## Conclusions

In conclusion, the perinatal period represents a critical phase in the establishment and development of the neonatal microbiome, with deep implications for long-term health. Factors such as mode of delivery, maternal diet, antibiotic exposure, and feeding practices significantly influence the composition and diversity of the infant gut microbiota. These early microbial communities are instrumental in shaping the neonate’s immune system, metabolism, and neurodevelopment. Table 1 summarizes the most important bacterial genera and species along with their associated functions and implications for health. This overview highlights the critical roles these microorganisms play during the perinatal period. The emerging evidence underscores the potential of targeted interventions, including dietary modifications, probiotic and prebiotic supplementation, and innovative practices like maternal vaginal seeding, to optimize microbiome development. However, further research is essential to fully understand the complex interactions between the perinatal microbiome and health outcomes and to develop effective strategies for promoting lifelong health starting from this pivotal period.

**Table 1:** Taxonomic classification of the main bacterial phyla, families, genera, and species involved in perianal period

Phylum	Family	Genus	Species	Function/association
Firmicutes	Lachnospiraceae	*Coprococcus*	/	“Obese-type” microbiota
		*Ruminococcus*	*R. gnavus*	“Obese-type” microbiota; increased in allergies
	Lactobacillaceae	*Lactobacillus*	/	Decreased in infants with colics; associated with breastfeeding
	Eubacteriaceae	*Eubacterium*	*E. rectale*	Involved in energy harvest, linked to obesity
	Clostridiaceae	*Clostridium*	/	Increase allergy risk; increased in formula-fed infant
	Staphylococcaceae	*Staphylococcus*	/	Increased in allergy; associated with C-section
Actinobacteria	Bifidobacteriaceae	*Bifidobacterium*		Associated with natural childbirth and breastfeeding; may decrease the risk of allergy
	Corynebacteriaceae	*Corynebacterium*	/	Associated with C-section
Proteobacteria	Enterobacteriaceae	*Escherichia*	*E. coli*	Associated with C-section
Bacteroidetes	Bacteroidaceae	*Bacteroides*	/	Increased in formula fed infant; decreased in CD-section
			*B. fragilis*	Decreased in obese children
Verrucomicrobia	Akkermansiaceae	*Akkermansia*	*A. muciniphila*	May decrease the risk of allergy

## Data Availability

No datasets were generated or analysed during the current study.
